# Commentary: Sleep Disturbance in Bipolar Disorder: Neuroglia and Circadian Rhythms

**DOI:** 10.3389/fpsyt.2021.730360

**Published:** 2021-09-30

**Authors:** Mauro Giovanni Carta, Daniela Fanni, Germano Orrù, Gavino Faa

**Affiliations:** ^1^Dipartimento di Scienze Mediche e Sanità Pubblica, Facoltà di Medicina e Chirurgia, Università di Cagliari, Cagliari, Italy; ^2^Department of Surgical Sciences, University of Cagliari, Cagliari, Italy

**Keywords:** bipolar disorder, thymosin beta-4, neurosteroids, social rhythm, biorhythm

## Introduction

Some contributions recently published on Frontiers in Psychiatry highlighted that dysregulation in daily rhythms could be related to higher risk of bipolar disorders (1, 2). On the other hand, dysregulation of sleep-wake rhythms and light pollution were found associated with prostate cancer (3). Indeed, it has been hypothesized the dysregulation of rhythmicity of melatonin and cortisol, the true markers of hormonal cycles, can affect the natural fluctuations of sex hormones, whose alteration could be the determinant of the increasing cancer risk (4, 5). An alteration in the concentrations of progesterone-derived hormones in blood has been identified during the luteal phase of the menstrual cycle in mood disorders with a concentration gradient that rises from healthy women, to women with stabilized depressive disorder and women with stabilized bipolar disorder, the latter with higher progesterone derived hormones level (6). Numerous steroids including those derived from progesterone have a specific effect and are synthesized in the Central Nervous System (neurosteroids) (7), in fact today they represent a horizon for research on the therapy of mood disorders (8–10). It was therefore hypothesized that progesterone could have an endogenous stabilizing role in contrast with the mood stimulating role of androgens, estrogens, and progestogens sulfates (8). It was argued that the alteration of circadian rhythms typical of bipolar disorders could have a link with the well-known rhythm's alterations of sex hormones (6, 8). It is also well-known that steroid and neurosteroid hormones have an active role in neuroplasticity, and that this aspect is strongly compromised in chronic mood disorders (11).

## Subsections Relevant for the Subject

Thymosin Beta-4 is a small protein involved in cell motiliy and tumorigenesis (12, 13). Thymosin beta-4 blood levels were found to be altered in both depressive and bipolar disorders. As such, the candidate role of Thymosin beta-4 as biomarker would deserve further investigation (14). It is known that thymosin beta 4 can affect the hypothalamus pituitary and medullary axis of the adrenal gland, with consequences on sex hormones (14). Thymosin beta 4 has in fact had been hypothesized to be a specific positive regulator of estrogen and negative regulator of progesterone derived hormones (15, 16). Furthermore, in a disorder such as toxoplasmosis, suspected to be associated with bipolar disorder (17–19), the vesicles produced by toxoplasma in neurological locations have been found to contain large amounts of thymosin beta4 (20).

## Discussion

All these indications suggest a possible link between timosine beta4, dysregulation of circadian rhythms, neurosteroid hormones and consequent high risk of mood and bipolar disorders. There is a need for future studies that focus on such evidence and hypotheses.

## Author Contributions

All authors listed have made a substantial, direct and intellectual contribution to the work, and approved it for publication.

## Conflict of Interest

The authors declare that the research was conducted in the absence of any commercial or financial relationships that could be construed as a potential conflict of interest.

## Publisher's Note

All claims expressed in this article are solely those of the authors and do not necessarily represent those of their affiliated organizations, or those of the publisher, the editors and the reviewers. Any product that may be evaluated in this article, or claim that may be made by its manufacturer, is not guaranteed or endorsed by the publisher.
